# Postbiotic Supplementation for Children and Newborn’s Health

**DOI:** 10.3390/nu13030781

**Published:** 2021-02-27

**Authors:** Daniela Morniroli, Giulia Vizzari, Alessandra Consales, Fabio Mosca, Maria Lorella Giannì

**Affiliations:** 1 Department of Clinical Sciences and Community Health, University of Milan, 20122 Milan, Italy; daniela.morniroli@unimi.it (D.M.); giulia.vizzari@unimi.it (G.V.); alessandra.consales@unimi.it (A.C.); maria.gianni@unimi.it (M.L.G.); 2 Fondazione IRCCS Ca’ Granda Ospedale Maggiore Policlinico, NICU, 20122 Milan, Italy

**Keywords:** microbiota, postbiotics, paraprobiotics, nonviable bacteria, gut axis

## Abstract

It is now well known how the microbiota can positively or negatively influence humans health, depending on its composition. The microbiota’s countless beneficial effects have allowed it to be defined as a genuine symbiont for our species. In an attempt to positively influence the microbiota, research has focused on probiotics and prebiotics. Probiotics are viable beneficial bacteria of various strains. Prebiotics are specific substances able to favor the development of advantageous bacteria strains. Postbiotics are a new category of compounds capable of affecting the microbiota. According to the different definitions, postbiotics include both nonviable bacteria and substances deriving from bacterial metabolism. Postbiotics are particularly promising in pediatric settings, as they offer some advantages over probiotics, including the absence of the risk of intestinal translocation or worsening of local inflammation. For these reasons, their use in fragile population categories such as newborns, and even more prematures, seems to be the best solution for improving microbiota’s health in this population. This narrative review aims to collect the research conducted so far on postbiotics’ potential in the first stages of life.

## 1. Introduction

### 1.1. The Role of Microbiota in Adult and Children’s Health

The discovery of the intestinal microbiota is among the most important ones of the last 50 years. From the first published work on gut bacteria, a considerable amount of research has described how the microbiota develops after birth, how it changes over time and, above all, what are the characteristics of a healthy microbiota [1].

Contrary to what was previously believed during fetal life, the uterine environment already contains bacteria of maternal origin. These microorganisms come into contact with the fetus through the amniotic fluid and begin the dialogue with its intestine. The maternal microbiota “trains” the fetus’ neonatal immune system to tolerate commensal microorganisms [2]. After birth, the newborn’s intestine is colonized by specific bacterial strains, which can originate from the mother or the surrounding environment, depending on the type of birth. Therefore, it appears evident that the mother’s microbiota conditions that of the child. The type of breastfeeding also influences the bacterial strains of the microbiota [3]. Spontaneous birth and breastfeeding favor the development of bacterial strains such as Lactobacilli and Bifidobacteria, among others, forming a “healthy” microbiota. These “healthy” commensal bacteria interact with the host organism, promoting immunological tolerance, thus decreasing the risk of inflammation and acting directly on the intestinal ecosystem, always with immunomodulatory and anti-inflammatory action [4]. Suppose the newborn’s intestine is colonized by bacterial strains considered harmful, including Clostridia and Enterococci. In that case, there will no longer be a positive dialogue with the child’s intestine, but rather an inflammation, low tolerance, and various types of pathologies, including allergies [2]. The microbiota continues to change even after the neonatal period, depending on various factors, including geographic location, maternal diet, weaning, diet, until it assumes its final form only in late adolescence [5]. Researchers then focused on the possibility of intervening to correct a microbiota defined as hostile. In fact, as in many other conditions, childhood represents a window of opportunity, where the action of the favourable microbiota translates into protection against specific pathologies that manifest in later ages [2]. It is therefore essential not to lose this time window when trying to influence the microbiota positively.

Research then expanded to demonstrate microbiota’s presence on other epithelial surfaces of the human body. This multitude of microorganisms plays a role of such importance for human health that it is considered, in its totality, a whole symbiotic organism, evolved together with our species with a mutual benefit. The presence of some particular microorganisms at the level of the human body’s epithelia would favor that particular district’s health [6,7]. Respiratory microbiota, ocular microbiota or vaginal microbiota are a few examples of this functional interaction. However, it is now clear that these beneficial effects of favourable microorganisms can act at a distance. A hostile intestinal microbiota has been associated with numerous diseases such as asthma and allergies, obesity, type 2 diabetes mellitus, a wide variety of psychiatric disorders and many others [8].

Some axes of communication between the intestine and specific target organs have been hypothesized to explain these remote effects. Among the most described, we find the intestine-brain axis. It has been hypothesized that the microbiota can influence some neurotransmitters’ production at the intestinal level, including serotonin. This effect would then be reflected on the central and peripheral nervous system, suggesting the reason for some clinical observations, such as the frequent coexistence of inflammatory bowel diseases with mood disorders [9]. The gut-brain communication axis appears to be how the gut microbiota manages to modulate neuronal activity. Among others, the gut-lung axis has also stimulated great interest. Researchers have suggested that the gut microbiota may promote the lung defenses, to control viral respiratory infections [10].

Considering the importance of the microbiota’s positive effect on human health, research has also focused on the possibility of promoting the development or restoration of healthy bacterial strains through the use of probiotics or prebiotics. Probiotics have been defined as live microorganisms which, when administered in adequate amounts confer a health benefit on the host [11]. Probiotics supplementation aims at introducing bacterial strains with a distinct and strain-specific beneficial effect. On the other hand, prebiotics are defined as “a selectively fermented ingredient that results in specific changes in the composition and activity of the gastrointestinal microbiota, thus conferring benefits upon host health” [12]. This definition, modified in 2008 by the International Scientific Association of Probiotics and Prebiotics (ISAPP), considers prebiotics as nondigestible compounds belonging mainly to the carbohydrate category and all other substances having the ability to influence the intestinal microbiota positively [13]. Therefore, the next reasonable step was to evaluate the combined effect of probiotics and prebiotics concomitantly, assuming a synergistic effect that would enhance each compound’s benefits. The mixture of probiotics and prebiotics in the same product is a synbiotic.

Synbiotics are defined as synergistic mixtures of probiotics and prebiotics that beneficially affect the host by improving the survival and colonization of live beneficial microorganism in the host’s gastrointestinal tract [14]. Synbiotics modulate both the gut microbiota composition and its metabolite production.

Hand in hand with discovering bacterial strains beneficial to human health and substances capable of promoting these strains’ proliferation, researchers have also focused on substances that could mediate the microbiota’s beneficial effects.

In recent years, numerous articles in the literature have shown that having a vital probiotic to have a beneficial effect may be unnecessary [15]. This has led to an increasing number of studies demonstrating the beneficial effects of administering nonviable microorganisms or bacterial metabolism products. This third category of beneficial products for humans’ health is commonly referred to as “postbiotic” [16].

The aim of this narrative review is to describe the state of the art in postbiotic research with a particular focus on their use in infancy and childhood.

### 1.2. The Discovery of Postbiotics

**Figure 1:**
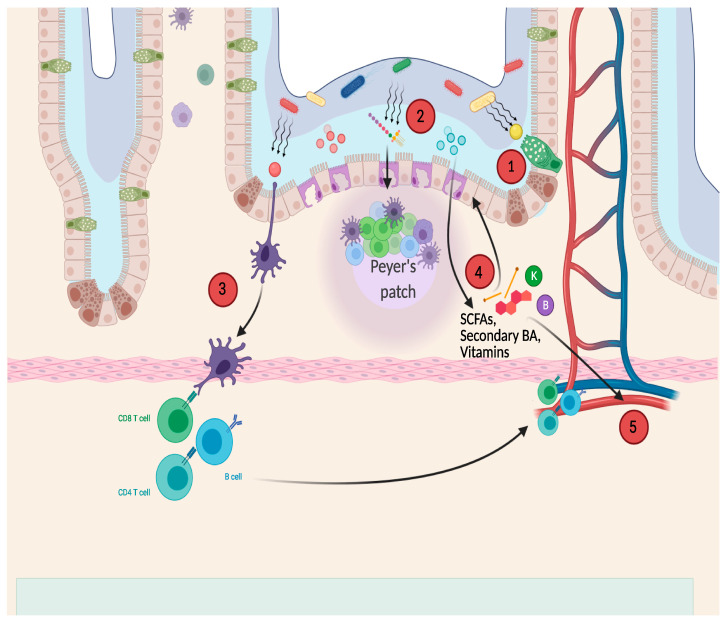
Main hypothesized pathways of postbiotic’s beneficial effects. 1, thickening of the mucus layer; 2-3, antimicrobial effect; 4, local anti-inflammatory effect; 5, systemic effects of postbiotics entering circulation. SCFAs: short-chain fatty acids; BA: biliary acids.

Although some studies have focused on postbiotics intended exclusively as byproducts of bacterial metabolism [17], to date, there is still no agreement among all researchers on which substances can be defined as postbiotics. Some authors enlist paraprobiotics, defined as “non-viable or inactivated microbial cells”, as a subgroup of postbiotics [16]. The other large group of postbiotics comprises a variety of compounds derived from the fermentation and metabolisation of various substances, performed by specific bacterial strains. Other authors consider nonviable bacteria not definable as a postbiotic, limiting the definition only for products of bacterial metabolism [18]. Postbiotics have been identified as part of the pathway that allows the intestinal microbiota to act both locally and at a distance, through the axes of communication between the intestine and the target organs [8]. To fully understand the postbiotic’s mode of action, it is essential to understand the intestinal surface structure and the layers in contact with gut bacteria (Figure 1).

The intestinal epithelium provides the first physical barrier against microorganisms in the gut lumen. This barrier comprises the mucus layer, the glycocalyx of intestinal epithelial cells, and the cell’s tight junctions. The mucus is a gel-like structure characterized by mucins’ presence, large, glycosylated structures secreted by the goblet cells. The mucus’ outer layer is covered with a multitude of intestinal microbes, while the inner layer, in close contact with the intestinal epithelium, does not contain microbes. The inner layer is, in fact, the real protective barrier against bacterial adhesion and invasion. It is now clear that the two layers of mucus, together with the intestinal epithelial cells, mediate the signaling between the gut and host immune cells by transferring mediators such as cytokines, chemokines and peptides [17].

Locally, postbotics have an immunomodulatory, anti-inflammatory, trophic and antimicrobial effect. One of the first local effects to be described is stimulating the intestinal epithelium cells to increase mucin production, thus improving the intestinal barrier. Postbiotics effects at a local level include the ability to reduce inflammation, interact with lymphocyte sites, modulate IgA’s immunity and production, and favor good bacterial strains [19].

A review by Mayorgas et al. listed the main substances defined as postbiotics and their beneficial effects on the gut epithelium and immune function [20]. To better understand these substances’ characteristics, the review authors have listed postbiotics according to their source. Among the postbiotics produced by the fermentation of substances introduced with the diet, great importance has been given to short-chain fatty acids (SCFAs), produced by the Bacteroidetes and Firmicutes strains and major end products of gut microbiota. SCFAs have been extensively studied for their anti-inflammatory properties in the intestine and their ability to favor the development of beneficial bacterial strains in a virtuous circle [21]. SCFAs, mainly acetate, propionate, and butyrate, are produced from the fermentation of prebiotics ingested with the diet and are known to act both at local and systemic levels [18]. Acetate and propionate are known to be adsorbed in the gut and enter the circulation, reaching muscles and other tissue. In particular, propionate is uptaken by the liver. Butyrate acts as an energy source for enterocytes in lower concentration, but inhibits the cell cycle in higher concentrations [22]. Locally, SCFAs are being studied for their powerful anti-inflammatory action, which has been exploited in inflammatory bowel diseases. They also are known to favor the development of the intestinal barrier and to contrast the development of colon’s cancerous cells [23].

Amongst the substances produced de novo by the intestinal microbiota with an apparent positive effect, we find bacterial polysaccharides. These substances produced by Bacteroidetes and Clostridium strains can stimulate cytokines with anti-inflammatory action. Two categories of de novo and perhaps lesser-known postbiotics are vitamins (B and K) and ATP. Both of these postbiotics can modulate intestinal immunity and ensure energy production. Postbiotics acting locally also include substances produced by the human body and subsequently modified by the intestinal microbiota. The secondary biliary acids generated by the intestinal lumen’s bile salts favor the epithelial barrier’s integrity and favor anti-inflammatory cytokines [20].

The most intriguing action of the postbiotics, however, is their remote action. Aguilar-Toalà et al. have published a recent paper to summarize the long-term beneficial effects of postbiotics: antioxidant, antiproliferative, hypocholesterolemic, antihypertensive and antiobesogenic [19].

The mechanisms by which postbiotics are able to exert their influence at a distance are not yet fully understood, as most of the studies have been performed in vitro. However, some studies have hypothesized possible metabolic pathway [24]. For example, their positive effect on lipid metabolism could be due to the activation of pathways inducing beta-oxidation of fatty acids and lipolysis in adipocytes. The reduction of hepatic insulin resistance and the activation of transcription factors that regulate glucose intolerance and inflammation of the adipose tissue appear to be the mechanism underlying the protective effect of postbiotics against obesity. This research area is promising, and future studies will bring new insight into these mechanisms, giving the possibility to exploit certain compounds and pathways to promote human health.

## 2. Postbiotics in Childhood

### 2.1. Advantages of Postbiotics Compared to Probiotic Use in Children

The pediatric population, especially the neonatal population, appears to be extremely appealing, when studying postbiotics’ positive effects. As already known, the gut at birth is still rather immature, both in its digestive and immune functions and in its barrier integrity. This translates into a potential greater intestinal reactivity and permeability which, in turn, if not kept under control by a favorable microbiota, can lead to a pro-inflammatory environment and immune dysregulation of various degrees [2].

Some studies have suggested that the intestinal microbiota evolves well beyond the first three years of life, seizing its final composition only after puberty [5]. 

Thanks to the symbiotic action between the favorable colonizing bacteria, the intestinal epithelium and the associated lymphoid system, the infant’s intestine matures after birth, improving its barrier functions and immunological regulation through the first stages of life.

As already mentioned above, postbiotics carry out a local anti-inflammatory, immunomodulating and antimicrobial action, against pathogenic strains, thus proving to have utmost interest in pediatric age, when intestinal immaturity predisposes to problems of this type.

Recent studies have investigated the possible pathways that lead to these beneficial effects. As already mentioned above, SCFAs and postbiotics have an essential role in promoting the intestinal barrier’s integrity, the protective mucus layer’s production, and above all, have an anti-inflammatory effect [18]. These three actions’ synergy is of great importance for the pediatric and neonatal population, where damage to the intestinal barrier and pro-inflammatory dysregulation underlied many pathologies of this age. Peptidoglycans have also been studied for their beneficial effects, with potential application in the pediatric age. A study by Clua et al. on a mouse model demonstrated how the intranasal administration of peptidoglycans derived from L. Rhamnosus has beneficial effects on the resistance to viral and bacterial respiratory infections [25]. In particular, the authors investigated those effects in Syncytial virus infection and secondary pneumococcal pneumonia, two of the main infective issues in the pediatric population worldwide.

It is now well known that intestinal bacteria play a role in producing vitamins that are important for human health. In particular, numerous bacterial strains present in the intestinal lumen can produce folate and B vitamins [26]. Folate has a paramount role in children’s anemia prevention, and other B vitamins are essential for a variety of metabolic pathways, mainly energy production.

A study by Prete et al. also described the potential beneficial effects of secondary bile acids, considered real postbiotics as the intestinal microbiota modifies them [27]. The bile acids metabolized by some bacterial strains activate a receptor response capable of modulating lipid metabolism, energy expenditure and therefore the growth and addition of fat mass. It seems that the bile acids modified by the microbiota can also modify the circadian rhythm, always correlating with the food intake and obesity risk. Obesity is a growing issue in the pediatric population worldwide, and postbiotics could be a promising supplement to fight against this global epidemic.

Postbiotics have significant safety advantages over probiotics [28]. Their use as supplements in critically ill patients, such as prematures, does not pose risks in terms of bacterial translocation and induction of bacterial resistance. Furthermore, it has been hypothesized that the use of probiotics in patients with diseases that lead to severe intestinal inflammation may even aggravate inflammation. When the intestinal ecosystem is compromised, even harmless bacterial strains can trigger the inflammatory response [17].

This is particularly valuable in a population at high risk such as premature babies, where the intestinal barrier’s integrity is impaired, the immune function is immature, and the clinical conditions are often critical. Furthermore, their use does not alter the development and physiological modification of the intestinal microbiota in various childhood milestones, especially in newborns. From these observations, in recent years, many scientists have rediscovered the functional value of fermented foods. In particular, regarding the pediatric population, they focused on the so-called FIFs or fermented infant formulas [29]. Understanding the unique role of breast milk in regulating the intestinal microbiota, research has focused in the attempt to reproduce part of the beneficial effect of breast milk, by adding compounds deriving from recognized bacteria strains in a healthy microbiota. 

### 2.2. Main Areas of Application for Postbiotics in Childhood

A review published by Wegh and colleagues has collected the main studies carried out in vivo in the pediatric population, classifying them according to the bacterial strain used in the fermentation process [30] (Table 1). It is important to remember that postbiotics’ positive effects on human health, are strictly dependent on the bacterial strain used for the fermentation process and the variety of food fermented.

Most of the studies have compared the benefits of using a FIF compared to a standard formula. Some studies have instead used supplements containing specific inactivated bacterial strains, with or without their culture medium, compared to placebos or prebiotics alone [30].

Most of the in vitro studies have investigated the possible positive effect of postbiotics in a population of healthy term infants. Two studies have employed cow’s milk fermented with L. paracasei CBA L74. The first demonstrated that the L. paracasei CBA L74 fermented food had a positive effect in shaping a favorable microbiota [32]. Subsequently, the authors described how this effect lead to an increased Butyrate synthesis.

The second study was a randomized controlled trial by Corsello et al. The authors investigated the effects of L. paracasei CBA L74 fermented milk on the incidence of common infections as a clinical outcome. The authors concluded that children taking the postbiotic supplement recorded fewer infections, both respiratory and gastrointestinal [31]. Other studies conducted on healthy term infants have tested the effect of various Lactobacillus strains, with or without the addition of prebiotics in various percentages (mainly FOS: Fructo-oligosaccharides and GOS: Galacto-oligosaccharides). These studies illustrate a positive effect of these by-products on intestinal function, represented by the incidence of colic disorders, the number and softness of stools and increased values of remarkable biomarkers of immune function, such as IgA levels [33].

Among the most investigated strains in the healthy infant population, we notice Bifidobacterium breve C50 and Streptococcus thermophilus 065. Postbiotics derived from these two strains, often used in combination for the product’s fermentation, have been shown to positively affect the antibody response after vaccinations, the markers of immune modulation and on the severity of episodes of intestinal infections. However, no effect was observed on the incidence of these infections [39,40].

Some studies have alternatively considered the effect of postbiotics on common childhood conditions, mainly diarrhea or allergic diseases.

Healing time in infants with non-rotavirus diarrhea was shortened by one day when taking lyophilized, heat-killed L. acidophilus LB plus their culture medium [34]. However, a study from Kaila et al., published in 1995 found no differences in infants’ clinical outcome treated with nonviable L. casei than viable units from the same strain [41]. 

Regarding allergic disorders, a study from Peng et al. proved that viable or heat-killed L. paracasei 33 bacteria had similar beneficial effects in children with perennial allergic rhinitis [35]. Investigating postbiotics in cow’s milk allergy and atopy, Morissett and Colleagues described the efficacy of infant formula with heat-inactivated with B. breve C50 and S. thermophilus in inhibiting the incidence of digestive and respiratory adverse events in infants at high risk of atopy. However, they failed to demonstrate an effect on cow’s milk allergy onset [36].

Interestingly, Kirjavainen et al. found no difference between live or heat-inactivated L. rhamnosus GG in atopic eczema and allergy to cow’s milk prevalence, also describing an association between heat-inactivated L. rhamnosus GG and adverse gastrointestinal symptoms [37].

Considering the studies cited above, the most studied bacterial strain in the production of postbiotics is Lactobacilli. Lactobacilli are gram-positive bacilli that include about 237 species and are among the most marketed and studied probiotics [42]. The first observations of the infant microbiota have shown various Lactobacilli strains in the gut since the first days of life [43]. A review by Tsegay et al. listed the various characteristics of Lactobacilli that explains their importance as postbiotics and paraprobiotics [44]. As paraprobiotics, their effect is expressed through the interaction between the host and the many proteins and substances on the various strains’ cell wall: teichoic acid, peptidoglycans, polysaccharides, and proteins belonging both to the cell membrane and the pili. Among Lactobacilli’s metabolism products with an outstanding postbiotic function we find SCFAs mentioned above; bacteriocins and conjugated linoleic acid with an immunomodulating and protective effect against pathogens, and aggregation-promoting factor, essential in promoting intestinal epithelial integrity. Remarkably, each Lactobacilli strain has a slightly different genomic asset, that differs from the others [42]. Therefore, each strain has a specific membrane and metabolic characteristics. This appears significant when supplementing probiotics or paraprobiotics in an attempt to improve a specific condition.

### 2.3. Postbiotics for Newborns and Prematures Health

As mentioned above, postbiotics could be a promising tool for newborns and prematures health. Current evidence supports the use of probiotic bacteria in this vulnerable population to prevent dreadful diseases such as necrotizing enterocolitis and late-onset sepsis. However, the risk of probiotic sepsis, intestinal translocation and transmission of antibiotic resistance, poses a hindrance to the use of probiotics in infants, especially if they are premature. A paper by Mosca et al. has hypnotized how postbiotics may be helpful for necrotizing enterocolitis prevention [22]. Considering the multifactorial pathogenesis of this disease, including intestinal dysbiosis; immaturity of the intestinal barrier; underdevelopment of adaptative and elevated expression of the Toll-like receptor 4, it appears reasonable that postbiotics could have a direct effect on most of these etiological factors. It has been described how postbiotics promote bacteriocins’ synthesis, substances capable of hindering the overgrowth of pathogenic bacteria.

Furthermore, it has now been demonstrated that some postbiotics, such as Butyrate, can promote the integrity of the intestinal barrier by promoting the production of the typical mucins of the intestinal epithelium (MUC2) [22]. Finally, a large number of postbiotics are known for their immunomodulatory efficacy, as described earlier in this review. 

Therefore, it appears evident the need to conduct studies aimed at analyzing the in vivo tolerability and efficacy profile of postbiotics in the most vulnerable population: premature babies. Very few studies have investigated in vivo the beneficial role of postbiotics in newborns health. A study by Campeotto et al. has compared the use of preterm formula fermented with B. breve C50 and S. thermophilus 065 with a standard preterm formula, describing a potential beneficial effect, demonstrated by lower fecal calprotectin levels and lower abdominal distention in the intervention group [38]. 

Paraprobiotics have also been studied as a safer alternative of probiotics in premature babies. In this regard, numerous in vitro and in vivo studies have investigated the potential risks and benefits of the administration of nonviable cells, demonstrating in general a good tolerability and safety profile of paraprobiotics but not concluding on their real effectiveness [16]. Similarly, a systematic review by Zorzela et al. has tried to give a more definite answer to the issue. Overall, from Zorzela’s work results, there would appear to be some evidence supporting the utilization of paraprobiotics in specific clinical situations. However, the study methods’ significant heterogeneity, the variety of bacterial strains investigated, and the small sample sizes of all studies do not support a more robust conclusion [45].

## 3. Conclusions

Postbiotics are indeed very promising supplements for human health. In particular, their positive effect on the development of the microbiota, intestinal maturity, and multiple immunomodulating actions makes them particularly interesting in children, as childhood is a crucial stage of opportunity for future health. In particular, postbiotics appear even more promising in neonates or premature babies, having a safety profile that would allow them to be used even in critically ill or extremely preterm infants. However, given the species-specific properties, the significant heterogeneity of the analyzed substrates and the great variability of potentially interfering conditions, to date, there are still no definite indications on their use in clinical practice. Further studies, primarily randomized controlled trials, will be needed to establish which bacterial strain efficiently produces beneficial postbiotics, their safety profile, recommended dosages and their advantageous effects in pediatric and neonatal disorders.

## Figures and Tables

**Table 1:** Main populations and conditions explored in postbiotics interventions studies in early life stages (Wegh et al., 2019) [30].

Study	Population	Age	Intervention	Comparison	Results
Corsello et al. 2017 [31]	Healthy, term children	12–48 months	Cow’s Milk Fermented with *L. paracasei* CBA L74	Cow’s milk with maltodextrin	Children presenting common infectious diseases were significantly lower in the intervention group. Significant changes in innate and acquired immune biomarkers were only observed in the intervention group.
Berni Canani et al. 2017 [32]	Healthy, term children	Cow’s milk powder Fermented with L. paracasei CBA L74	Cow’s milk powder with maltodextrin	12–48 months	The intervention, not placebo, showed an increase in the relative abundance of predicted genes involved in butyrate synthesis
Huet et al. 2017 [33]	Healthy, term infants	Lactofidus 50%FERM, scGOS/lcFOS+ 15%FERM or scGOS/lcFOS+ 50%FERM	scGOS/lcFOS	0–28 days	Infant colic was significant lower (8%) with scGOS/lcFOS + 50% FERM than scGOS/lcFOS
Lievin-Le Moal et al. 2007 [34]	Infants with acute diarrhea	Heat-killed *L. acidophilus* LB plus culture medium	Placebo	10 months	Recovery time of infants with nonrotavirus diarrhea was shortened by 1 day when taking lyophilized, heat-killed *L. acidophilus* LB plus their culture medium
Peng et al. 2005 [35]	Children with perennial allergic rhinitis	Capsules with live or heat-killed *L. paracasei* 33	Placebo	<18 years	Efficacy of heat-killed *L. paracasei* LP33 was not inferior to the live variant.
Morisset et al. 2011 [36]	Infants with a high risk of atopy	Infant formula, heat-inactivated with *B. breve* C50 and *S. thermophilus*	Standard infant formula	Birth	The fermented formula did not alter proportion of children with cow’s milk allergy, but decreased the proportion of positive skin prick tests to cow’s milk, incidence of digestive AEs, and respiratory potentially allergic AEs at 12 months
Kirjavainen et al. 2003 [37]	Infants with atopic eczema and allergy to cow’s milk	Infant formula containing live or heat inactivated *L. rhamnosus* GG	Hydrolyzed whey formula	Mean age 5.5 months	No differences were found in the bacterial numbers within the genera enumerated. However, heat inactivated *L. rhamnosus* GG was associated with adverse gastrointestinal symptoms and diarrhea.
Campeotto et al. 2011 [38]	Pre-term infants 30–35 weeks of GA	0–3 days	Preterm infant formula with heat-inactivated FERM with B. breve C50 and S. thermophilus 065	Preterm infant formula	No differences between groups in anthropometrics and digestive tolerance, except abdominal distention, which was lower in the FERM group (0 FERM vs. 8 control, *p* = 0.016). Significant lower fecal calprotectin was found in the FERM group from week 3
